# Comparative Analysis of Safety and Efficacy: Conventional Mediolateral Pinning vs. Dorgan’s Lateral Cross-Pinning in Pediatric Supracondylar Fractures

**DOI:** 10.7759/cureus.93662

**Published:** 2025-10-01

**Authors:** Muhammad Waqas Khan, Shayan A Irfan, Shahzeb Solangi, Radeyah Waseem, Muhammad Sheheryar Hussain, Syed Muhammad M Bin Sultan, Aleena Aftab, Anum Naqvi, Alina Fatima, Mahnoor Raza

**Affiliations:** 1 Trauma and Orthopaedics, Dow University of Health Sciences, Karachi, PAK; 2 Cardiology, National Institute of Cardiovascular Diseases, Karachi, PAK

**Keywords:** conventional mediolateral pinning, dorgan's lateral cross-pinning, pediatric orthopedics, pinning techniques, supracondylar fractures, ulnar nerve injury

## Abstract

Supracondylar fracture of the humerus (SFH) is a common occurrence in the developing skeleton, comprising a majority of all elbow injuries. Various pinning techniques have been proposed for displaced pediatric SFH, balancing the superior stability of cross pinning against its risk of ulnar nerve injury. The lateral and modified lateral cross-pinning approaches emerge as safer alternatives to prevent ulnar nerve injury. This study aims to compare the safety and efficacy of two pinning techniques, conventional mediolateral pinning and Dorgan's lateral cross-pinning, for treating Gartland type II, III, and IV supracondylar fractures in children. A systematic review and meta-analysis were conducted following Preferred Reporting Items for Systematic Reviews and Meta-Analyses (PRISMA) guidelines. PubMed/Medline, Cochrane Trial Register, and Google Scholar were searched for studies comparing the two pinning techniques. The inclusion criteria involved Gartland type II, III, and IV fractures in children, including randomized controlled or observational studies, with outcomes related to functional results and ulnar nerve impingement. Quality assessment was performed using the Newcastle-Ottawa Scale. Four studies with a total of 282 participants were included.

The Dorgan technique demonstrated comparable outcomes to mediolateral pinning in terms of carrying angle and range of motion. However, Dorgan's technique significantly reduced the risk of ulnar nerve impingement compared to conventional pinning (risk difference = -0.09, 95% CI = -0.13, -0.04, p = 0.0002). In the management of Gartland type II, III, and IV supracondylar fractures in children, Dorgan's lateral cross-pinning technique presents a safer alternative to conventional mediolateral pinning by significantly decreasing the risk of ulnar nerve injury. While both techniques offer comparable functional outcomes, clinicians should weigh the benefits of reduced nerve complications against potential radial nerve risks and longer treatment times associated with Dorgan's technique. Individualized decision-making considering patient factors is crucial in selecting the appropriate pinning method for optimal fracture management.

## Introduction and background

Supracondylar fracture of the humerus (SFH) is a common occurrence in the developing skeleton, comprising approximately 60% of all elbow injuries in the first decade of life [1]. It can be classified into flexion and extension types, the extension-type fractures being more prevalent, and further classified by Gartland into three major categories (type I, type II, and type III). Later, Leitch et al. included type IV, describing multidirectional instability (Table 1) [2].

**Table 1:** Gartland classification Sourced from *Management of Supracondylar Fractures of the Humerus in Children* by Gartland JJ [2].

Type	Description
Type I	Undisplaced or minimally displaced fracture with intact anterior humeral line.
Type II	Small deviation, fragments in contact with intact posterior cortex
Type III	Complete displacement of the fracture fragments and breach in the posterior cortex.
Type IV	Multidirectional instability

The most popular technique for surgical treatment of children with displaced SFH (type II, III, and IV) is closed reduction and stabilization with percutaneous pins [3]. In the literature, numerous pinning techniques have been proposed. Flynn and Swenson have utilized two cross pins placed medially and laterally, citing improved biomechanical stability. Nonetheless, this technique carries a risk of ulnar nerve injury in 2% to 8% of cases during medial pin insertion [4]. To prevent ulnar nerve injury, Arino et al. recommend lateral pinning with two pins mounted laterally. This method proves to be safer, but its biomechanical fixation stability in comparison to cross pinning is debatable [5]. In light of these considerations, Dorgan introduced a novel perspective on lateral pinning. His approach involves placing the pins laterally, but unlike conventional lateral pinning, the second pin is inserted from the lateral superior to the medial inferior. This lateral cross-pinning technique eliminates the risk of ulnar nerve injury and provides enhanced biomechanical stability [6]. This review intends to compare the safety and efficacy of two pinning techniques in Gartland type II, III, and IV to assess the comparative outcomes of the conventional method versus Dorgan's method regarding radiological and functional results and difficulties with fixing misaligned SFH in children.

This article was previously posted to the Research Square preprint server on November 27, 2024.

## Review

Method

Data Sources and Search Strategy

The Preferred Reporting Items for Systematic Review and Meta-analyses (PRISMA) guidelines were adopted for this systematic review and meta-analysis [7]. An electronic search from PubMed/Medline, Cochrane Trial Register, and Google Scholar was conducted from their inception till June 2025, using “Supracondylar humerus fracture,” “Medial-lateral cross-pinning,” “Lateral cross-pinning,” and “Dorgan's technique” as keywords. Manual screening of articles that were cited in any related meta-analysis, cohort studies, and review articles was conducted to identify any relevant studies. Our outcomes of interest were functional outcomes, including Flynn’s criteria for range of motion and carrying angle, and post-procedural ulnar nerve impingement [4]. Two authors individually evaluated all related records, and any disparities were resolved after mutual consent.

Study Selection

We employed the following screening method for the retrieved publications: combined title and abstract screening accompanied by full-text screening. The inclusion criteria included randomized controlled studies or observational studies with a population including children with extension-type SFH (Gartland type II, III, and IV fractures) comparing the Dorgan technique with the conventional (mediolateral cross-pinning) technique. We established the exclusion criteria as follows: Gartland type I fractures; fractures with concurrent upper limb injuries at distant sites; fractures with neurovascular compromise; and open fractures.

Data Extraction

Studies that met the criteria for inclusion underwent screening and evaluation by two separate reviewers. In an online accessible database entry software, we created sheets to collect all possible data from the selected studies, which included the total number of patients enrolled in the different groups, study type, mean age of patients, gender, classification of fracture as per the Gustilo-Anderson classification, and mechanism/cause of injury. Raw data for functional outcomes, including Flynn’s criteria for cosmetic angle and range of motion and postoperative ulnar nerve impingement, were recorded. Data for cosmetic angle and range of movement were compiled by combining data for excellent plus good and fair plus poor. 

Two reviewers independently searched all three electronic databases. The studies searched were exported to the EndNote Reference Library software version 20.0.1 (Clarivate Analytics, Philadelphia, PA, USA), and duplicates were screened and removed. Two investigators independently extracted data, entering them on a computer spreadsheet. Discrepancies were resolved through consensus discussions among investigators.

Quality Assessment of Studies

Three investigators independently assessed the quality of each included study. The Newcastle-Ottawa scale (NOS) [8] and Cochrane criteria for randomized controlled trials (RCTs) [9] were used to assess the quality of the cohort studies and RCTs, respectively. The NOS score <6 was considered a high risk for bias, 6-7 was moderate, and a score >7 was considered a low risk of bias.

Selected studies were comparative studies, and the NOS was used to perform quality assessment [8]. The NOS operates by classifying the study as good quality, fair quality, or poor quality based on the Agency for Healthcare Research and Quality (AHRQ) criteria [10]. To be classified as a good-quality study, the study needs to have 3 to 4 stars in the study selection domain, 1 to 2 stars in the comparability domain, and 2 to 3 stars in the outcome or exposure domain. To be categorized as fair quality, a study needs to have 2 stars in the study selection domain, 1 to 2 stars in the comparability domain, and 2 to 3 stars in the outcome or exposure domain. A poor-quality study needs to have 0 to 1 stars in the study selection domain, or 0 stars in the comparability domain, or 0 to 1 stars in the outcome or exposure domain [9].

Statistical Analysis

Review Manager (RevMan) version 5.4.1 (The Cochrane Collaboration, London, GBR) was employed for comparison of the Dorgan technique compared to conventional treatment. The data from studies were pooled using a random-effects model. Analysis of results was done by calculating the risk difference (RD) with respective 95% confidence intervals (CI). A p-value < 0.05 was considered significant for all analyses.

Results

Literature Search Results

**Figure 1:**
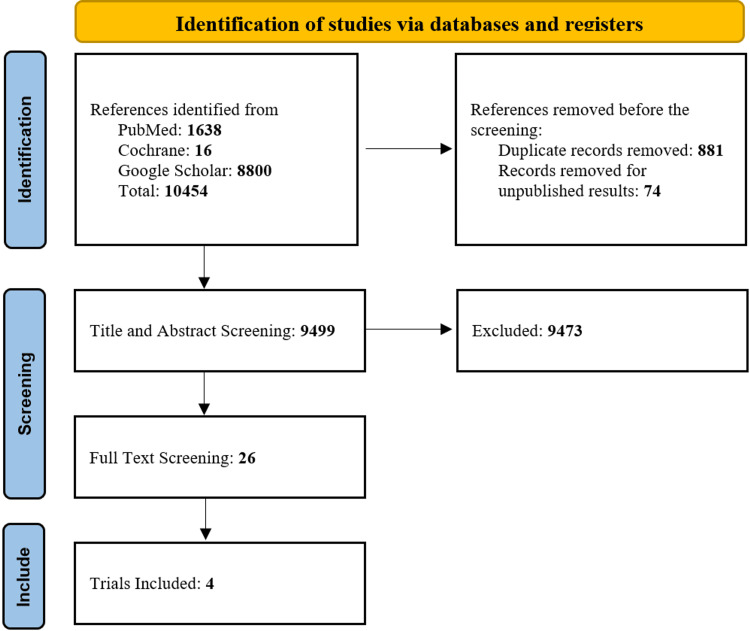
PRISMA flow diagram for systematic review and meta-analysis PRISMA: Preferred Reporting Items for Systematic Review and Meta-analyses [7]

The initial search of the three electronic databases yielded 10,454 potential studies, which were reduced to 9,499 after removal of duplicates. After exclusions based on titles and abstracts, the full texts of 26 studies were read for possible inclusion. Seven studies were excluded due to obscure methodology, six studies were excluded due to irrelevant and insufficient data, and three studies were not in English. A total of four studies remained for qualitative analysis and quantitative analysis [11-14]. Figure 1 summarizes the results of our literature search.

Study Characteristics

The four studies included 282 participants. Of these, 146 participants underwent the conventional method and 136 underwent the Dorgan technique to treat SFH. Tables 2-3 provide the demographic and clinical characteristics of the included studies.

**Table 2:** Demographic characteristics of patients in the included studies

Authors	Year of publication	Total no. of patients (n)	Conventional method (Group 1)	Dorgan method (Group 2)	No. of males, n (%)	No. of females, n (%)	Age (mean±SD)		Fracture type, n (%)			Mode of fracture, n (%)			Side affected, n (%)		Displacement, n (%)		
							Group 1	Group 2	Gartland IIa	Gartland IIb	Gartland III	Fall from height	Motor vehicle accident	Bicycle and game accident	Right	Left	Posteromdial	Posterolateral	Direct posterior
Ducic et al. [12]	2016	138	71	67	88 (63.7)	50 (36.2)	6.7±1.6	6.5±1.85	12 (8.6)	35 (25.3)	91 (65.9)	NA	NA	NA	59 (42.7)	79 (57.2)	NA	NA	NA
Rizk et al. [11]	2019	50	25	25	32 (54)	18 (36)	5.2±2.7	7.8±3.1	NA	NA	50 (100)	25 (50)	6 (12)	19 (38)	23 (46)	27 (54)	24 (48)	15 (30)	11 (22)
Othman et al. [14]	2017	47	17	14	NA	NA	5.32±2.92	6±2.29	22 (46.8)		25 (53.1)	NA	NA	NA	NA	NA	NA	NA	NA
Memisoglu et al. [13]	2011	139	75	64	114 (82.0)	25 (17.9)	7.5	7.8	NA	NA	139 (100)	87 (62)	31 (22)	21 (16)	43 (31)	96 (69)	38 (27)	79 (57)	17 (12)

**Table 3:** Clinical characteristics of the included studies

Study	Follow-up period	Fracture types	Cosmetic outcome (%)	Functional outcome (%)	Radiologic outcome	Nerve injuries (%)	Pin tract infection (%)	Time of procedure	Vascular complications	Neurological complications	Follow-up duration	Loss of reduction (%)
Othman et al. [14]	7±1.5 months	Supracondylar humeral fractures	93.75 (medial-lateral pinning)	87.5 (medial-lateral pinning)	Fractures united at 4±1.2 weeks	Iatrogenic ulnar nerve injuries	12.5 (medial-lateral pins)	NA	NA	4.44 iatrogenic ulnar nerve damage	Six to 10 months	No loss of reduction
Rizk et al. [11]	25.24±7.2 months	Gartland type III fractures	NA	NA	Fractures united at 5.9±1.3 weeks	NA	4.4 (Dorgan's method)	Significant difference (p=0.001)	Satisfactory collateral circulation	Not observed	11 to 36 months	No statistically significant difference
Ducic et al. [12]	11.2±2.3 months	Extension-type supracondylar	90 (standard pinning)	90 (excellent), 10 (good)	NA	No neurological complications	9.9 (standard pinning)	More time-consuming (Dorgan’s method)	Satisfactory collateral circulation	Not observed	Not specified	No statistically significant difference
Memisoglu et al. [13]	Three years (mean)	NA	NA	NA	NA	Iatrogenic ulnar nerve damage	No statistically significant difference	Significant difference (p<0.05)	NA	Not observed	No statistically significant difference	NA

Risk of Bias Assessment

The risk of bias was assessed using the Cochrane criteria for RCTs and NOS in accordance with AHRQ standards and was classified as good, fair, or poor [9,8,10]. All the included studies were reported to be of good quality (Tables 4-5).

**Table 4:** Quality assessment of included cohorts using the NOS NOS: Newcastle Ottawa scale [8]

Studies	Selection (maximum four)				Comparability (maximum two)	Outcome (maximum three)			Total score
	Representativeness of the exposed cohort	Selection of the non-exposed cohort	Ascertainment of exposure	Demonstration that the outcome of interest was not present at the start of the study	Comparability of cohorts based on the design or analysis	Assessment of outcome	Was the follow-up long enough for outcomes to occur?	Adequacy of follow-up of cohorts	
Memisoglu et al. [13]	1	1	1	1`	2	1	1	1	9
Othman et al. [14]	1	1	1	1	2	1	1	1	9

**Table 5:** Quality assessment of included trials using Cochrane criteria for RCTs RCTs: Randomized controlled trials; Cochrane criteria [9]

Study	Random sequence generation	Allocation concealment	Blinding (participants and personnel)	Blinding (outcome assessment)	Incomplete outcome data	Selective reporting	Other sources of bias	Net risk
Ducic et al. [12]	Low risk	Low risk	Low risk	Low risk	Low risk	Low risk	Unclear risk	Low risk
Rizk et al. [11]	Low risk	Low risk	Low risk	Low risk	Low risk	Unclear risk	Low risk	Low risk

Results of Meta-Analysis

In our meta-analysis, we assessed Flynn’s criteria for carrying angle, Flynn’s criteria for range of motion, and the incidence of ulnar nerve impingement [4].

**Figure 2:**
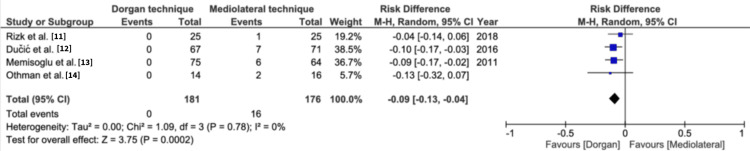
Flynn's criteria for carrying angle for excellent and good outcomes Flynn's criteria sourced from* Blind Pinning of Displaced Supracondylar Fractures of the Humerus in Children. Sixteen Years’ Experience With Long-Term Follow-Up *by Flynn et al. [4]. M-H: Mantel-Haenszel method

**Figure 3:**
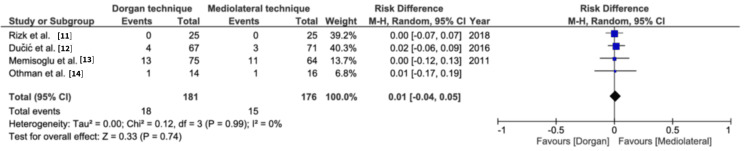
Flynn's criteria for carrying angle for fair and poor outcomes Flynn's criteria sourced from *Blind Pinning of Displaced Supracondylar Fractures of the Humerus in Children. Sixteen Years’ Experience With Long-Term Follow-Up* by Flynn et al. [4]. M-H: Mantel-Haenszel method

Flynn’s criteria for carrying angle: All four studies present the data for Flynn’s criteria for carrying angle. The good and excellent outcomes were pooled together and showed statistically nonsignificant differences between the Dorgan technique and mediolateral fixation (risk difference = 0.00, 95% CI=-0.05, 0.05, p=0.93) (Figure 2). The poor and fair outcomes pooled separately also indicated no statistical difference between the two techniques (risk difference = 0.01, 95% CI=-0.04, 0.05, p=0.74). There was no heterogeneity reported between the groups (Figure 3).

**Figure 4:**
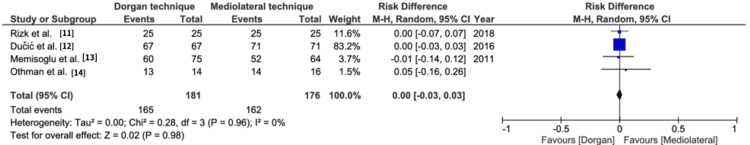
Flynn's criteria for range of motion for excellent and good outcomes Flynn's criteria sourced from *Blind Pinning of Displaced Supracondylar Fractures of the Humerus in Children. Sixteen Years’ Experience With Long-Term Follow-Up* by Flynn et al. [4]. M-H: Mantel-Haenszel method

**Figure 5:**
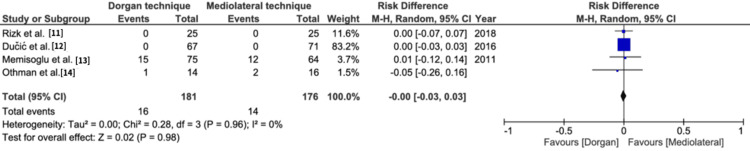
Flynn's criteria for range of motion for fair and poor outcomes Flynn's criteria sourced from *Blind Pinning of Displaced Supracondylar Fractures of the Humerus in Children. Sixteen Years’ Experience With Long-Term Follow-Up* by Flynn et al. [4]. M-H: Mantel-Haenszel method

Flynn’s criteria for range of motion: All four studies presented the data for the range of motion. Both techniques showed similar outcomes for good and excellent range of motion (risk difference= 0.00, 95% CI=-0.03, 0.03, p=0.98), and poor and fair range of motion (risk difference= 0.00, 95% CI=-0.03, 0.03, p=0.98) (Figures 4-5, respectively). Heterogeneity was not reported in the results.

**Figure 6:**
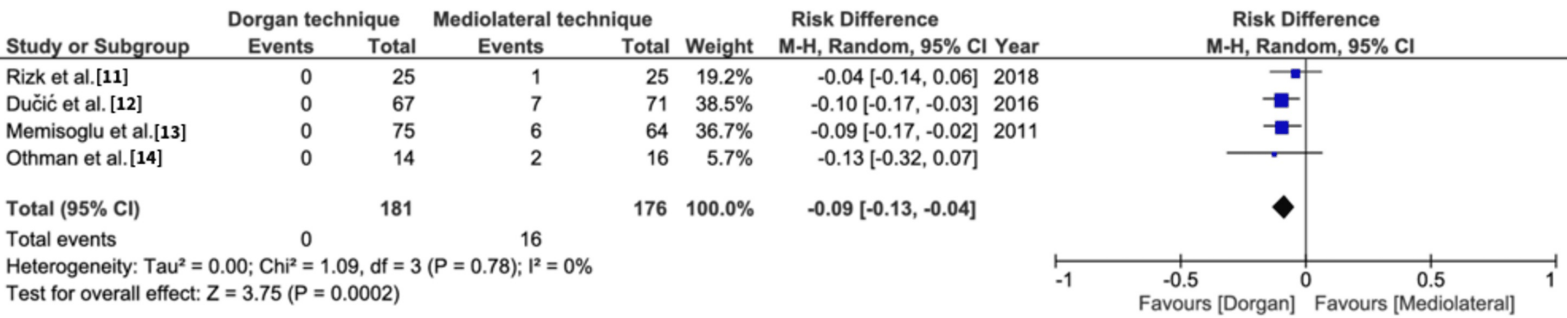
Ulnar nerve impingement M-H: Mantel-Haenszel method

Ulnar nerve impingement: This result was reported by all the included studies. The conventional Dorgan technique was found statistically superior to mediolateral fixation (risk difference=-0.09, 95% CI=-0.13, -0.04, p=0.0002). Heterogeneity was reported as 0% (Figure 6).

Discussion

The most adequate management of SFH in the pediatric population has been a topic of extensive research and clinical debate. The results of our meta-analysis provide a valuable insight into the comparison of conventional mediolateral fixation and the Dorgan technique. Our study corroborates the novel finding that the Dorgan technique is associated with a significantly reduced risk of iatrogenic ulnar nerve injury, which is a common complication in SFH management.

In their systematic review and meta-analysis, Marson et al. [15] evaluated the interventions for treating SFH in children, highlighting the surgical and non-surgical approaches, pinning techniques, and reduction methods. Their findings, based on 52 trials involving 3594 children, suggested that retrograde lateral wires may be associated with a lower risk of nerve injury compared to retrograde crossed wires, while open versus closed reduction may not significantly impact nerve injuries, but closed reduction may reduce major complications. However, the certainty of this evidence is generally low. The review highlights the need for further research, particularly with a core outcome set that includes patient-reported measures, to guide optimal management for these fractures in children and address the limitations in the available evidence.

The literature suggested significantly positive outcomes for types I and II clavicle fractures managed non-operatively. However, type III requires close reduction without any skin incision and percutaneous pinning approaches. Type IV needs to be managed operatively by open reduction and internal fixation [16,17]. Type III fractures are a surgical emergency to ameliorate the risk of complications, which involve compromise in the movement of the joint and cosmetic appearance [18-20]. Despite the established use of percutaneous Kirschner wire fixation for type III fractures, the approach is still debatable.

Carrazzone et al. drew a comparison between crossed versus lateral Kirschner (K)-wires to provide insight into the carrying angle, range of motion, and neurologic lesions. The study primarily assessed the safety of lateral wire fixation concerning neurologic injuries. The children who underwent crossed wire fixation had a higher risk of neurological injury, specifically ulnar nerve injuries [21]. Kwok et al. corroborated the findings of Carrazzone et al. regarding loss of reduction and iatrogenic ulnar nerve injury, indicating that lateral pinning is more favorable in these aspects [21,22]. However, we found the literature lacking in drawing a comparison between the mediolateral technique and the Dorgan techniques.

Kirschner wires are metal wires inserted into the bone for support while it heals. Approaches commonly employed are the medio-lateral cross-wire technique, lateral-wire technique, and lateral crossed-wire or Dorgan technique [20,21]. Fracture stability is deemed more pronounced in cross-wire techniques than lateral pinning; however, a retrospective review conducted by Queally et al. found that Dorgan is equally effective in maintaining stability as traditional cross-wiring [23]. Another common complication is iatrogenic ulnar nerve injury, with a reported incidence of 2% to 8% [24]. The incidence has been associated with mediolateral crossed-pinning in a review by Topping et al. [25]. A significantly lower tendency has been observed when wires are inserted laterally, as concluded by a systematic review of 33 studies by Brauer et al. [26]. The claim is also supported by the results obtained in our meta-analysis, which show that lateral cross-wiring is also associated with a considerably reduced risk of iatrogenic ulnar nerve injury.

Despite the comparable stability and lower risk of iatrogenic injury, the Dorgan technique can potentially impact the radial nerve. The anatomical location of the radial nerve, which lies anterior to the lateral intermuscular septum, makes it liable to injury by the proximal wire, which enters 2 cm away from the nerve. This makes the radial nerve susceptible to injury when it lies at the lower third of the humerus, under the brachioradialis [24]. This phenomenon can be overcome by directing the insertion of the wire relatively posterior to the mid-coronal plane. Care is also suggested during conventional cross-wiring, where wire insertion from the medial condyle is halted 2 mm away from the cortex to avoid impingement of the ulnar nerve. The impending risk is accentuated by a case report by Gangadharan et al., where the K-wire entering 1.5 cm proximal to the lateral condyle compromised the radial nerve [27].

Pin-site infections are reported in cases ranging from 1% to 21%, owing to the fact that the ends of the pins lie exterior to the skin [28]. A study performed by Parikh et al. investigated the contributory factors for these infections and established that a higher rate was noted in intracapsular pins. This association was further elaborated to state that parallel pins traversed through the joint capsule to a higher degree than cross-pins and thus were more susceptible to infection [29]. Other factors that merit mentioning are that pin size may correspond to the child’s body weight, where a 20 kg weight may be used as a threshold. Pins measuring 2 mm could be opted for if the child is above 20 kg, while a 1.5 mm size is appreciated for children under 20 kg. Another notable finding described by Rizk et al. is the prolonged treatment time consumed by Dorgan’s technique compared to other approaches [11].

However, the Dorgan technique is not without its challenges. It is worth noting that while it provides comparable stability and a lower risk of ulnar nerve injury, it can potentially jeopardize the radial nerve. This limitation stems from the anatomical proximity of the radial nerve to the lateral intermuscular septum. To mitigate this risk, wire insertion must be directed relatively posterior to the mid-coronal plane [30]. It is crucial for surgeons to exercise care and precision during wire insertion, regardless of the chosen technique, to prevent nerve injuries. Additionally, pin-site infections are a common complication, with rates ranging from 1% to 21%. The choice of pins and their size in relation to the child's body weight is crucial, with smaller pins recommended for lighter children. Moreover, the approach to pin insertion, whether intracapsular or cross-pinning, has been associated with varying infection rates [11,13]. It is essential to recognize that despite comparable stability and reduced iatrogenic nerve injury risk, the Dorgan technique might necessitate longer treatment times compared to other approaches [16]. This is an important consideration for clinicians when choosing the most suitable approach for their patients.

While our systematic review and meta-analysis aimed to provide valuable insights into the comparison between the Dorgan technique and the mediolateral technique for treating SFH, there are several notable limitations that should be considered. The assessment of publication bias was hindered due to the relatively small number of included studies, which limited our ability to comprehensively evaluate the potential for publication bias. Furthermore, our strict inclusion criteria, which focused on randomized controlled trials and observational studies, may have excluded other relevant study designs, potentially leading to a degree of selection bias. It is essential to acknowledge the variations in the methodological quality of the included studies, as they may introduce bias into our results. Additionally, our analysis was based on a relatively small sample size. This limited sample size may impact the generalizability of our findings and increase the risk of type II errors. The presence of substantial heterogeneity among the included studies was observed, which, if not thoroughly addressed, could have affected the validity of the pooled results. Variability in the surgical techniques employed across the included studies, as well as potential differences in the execution of the Dorgan technique and conventional methods, may introduce additional sources of bias. We also acknowledge that, despite our efforts to search comprehensively, language bias may have been introduced since our search was limited to English-language publications.

The study has limitations that warrant consideration in interpreting its findings. A relatively small sample size across included studies may constrain the generalizability of results, emphasizing the need for a larger and more diverse sample for enhanced statistical robustness. Assessment of publication bias was hindered by the limited number of studies, potentially leaving the study vulnerable to the influence of unpublished or overlooked research with differing outcomes. Language bias is a concern, given the study's exclusive focus on English-language publications, which may potentially exclude relevant studies in other languages. Methodological variations in surgical techniques and potential differences in the execution of Dorgan's technique and conventional methods may introduce bias and affect comparability. Substantial heterogeneity among studies, stemming from differences in design, patient populations, and surgical expertise, could impact the validity of pooled results. Incomplete reporting of data, strict inclusion criteria, and variations in study quality introduce further limitations. Despite these constraints, the study offers valuable insights into pinning techniques for pediatric SFH, highlighting the importance of careful interpretation within the context of these limitations.

## Conclusions

Our systematic review and meta-analysis provide valuable insights into the management of SFH, particularly concerning the choice of pinning techniques. While our findings support the advantages of the Dorgan technique in terms of reduced ulnar nerve injury risk, it is crucial for surgeons to remain vigilant regarding potential radial nerve complications. Additionally, the choice of pin size and insertion approach should be tailored to the patient's specific needs. Our study underscores the complexity of decision-making in the surgical management of these fractures and highlights the need for a nuanced, patient-centered approach in clinical practice.

## References

[1] Hubbard EW, Riccio AI (2018). Pediatric orthopedic trauma: an evidence-based approach. Orthop Clin North Am.

[2] Gartland JJ (1959). Management of supracondylar fractures of the humerus in children. Surg Gynecol Obstet.

[3] Dalrymple J, Ahluwalia A, Prinja A (2024). Paediatric supracondylar fractures: assessment and management. Br J Hosp Med.

[4] Flynn JC, Matthews JG, Benoit RL (1974). Blind pinning of displaced supracondylar fractures of the humerus in children. Sixteen years' experience with long-term follow-up. J Bone Joint Surg Am.

[5] Ariño VL, Lluch EE, Ramirez AM, Ferrer J, Rodriguez L, Baixauli F (1977). Percutaneous fixation of supracondylar fractures of the humerus in children. J Bone Joint Surg Am.

[6] Shannon FJ, Mohan P, Chacko J, D'Souza LG (2004). "Dorgan's" percutaneous lateral cross-wiring of supracondylar fractures of the humerus in children. J Pediatr Orthop.

[7] Hutton B, Salanti G, Caldwell DM (2015). The PRISMA extension statement for reporting of systematic reviews incorporating network meta-analyses of health care interventions: checklist and explanations. Ann Intern Med.

[8] (2021). Ottawa Hospital Research Institute. https://www.ohri.ca/programs/clinical_epidemiology/oxford.asp.

[9] Cumpston M, Li T, Page MJ, Chandler J, Welch VA, Higgins JP, Thomas J (2019). Updated guidance for trusted systematic reviews: a new edition of the Cochrane Handbook for Systematic Reviews of Interventions. Cochrane Database Syst Rev.

[10] AHRQ - quality indicators. https://qualityindicators.ahrq.gov/measures/psi_resources.

[11] Rizk AS, Kandil MI (2018). Conventional versus lateral cross-pinning (Dorgan's technique) for fixation of displaced pediatric supracondylar humeral fractures: a randomized comparative study. Egypt Orthop J.

[12] Dučić S, Radlović V, Bukva B (2016). A prospective randomised non-blinded comparison of conventional and Dorgan's crossed pins for paediatric supracondylar humeral fractures. Injury.

[13] Memisoglu K, Cevdet Kesemenli C, Atmaca H (2011). Does the technique of lateral cross-wiring (Dorgan's technique) reduce iatrogenic ulnar nerve injury?. Int Orthop.

[14] Othman M, Nahla A, El-Malt El-Malt (2017). A comparative study of three percutaneous pinning techniques for paediatric supracondylar humeral fractures. ARC J Orthop.

[15] Marson BA, Ikram A, Craxford S, Lewis SR, Price KR, Ollivere BJ (2022). Interventions for treating supracondylar elbow fractures in children. Cochrane Database Syst Rev.

[16] Booker M, Sumandea F, Pandya N, Swarup I (2025). Nonoperative management of Gartland type II supracondylar humeral fractures: a comprehensive review. Curr Rev Musculoskelet Med.

[17] Lim KB, Lim CT, Tawng DK (2013). Supracondylar humeral fractures in children: beware the medial spike. Bone Joint J.

[18] Kocher MS, Kasser JR, Waters PM (2007). Lateral entry compared with medial and lateral entry pin fixation for completely displaced supracondylar humeral fractures in children. A randomized clinical trial. J Bone Joint Surg Am.

[19] Shrader MW (2008). Pediatric supracondylar fractures and pediatric physeal elbow fractures. Orthop Clin North Am.

[20] Anari JB, Arkader A, Spiegel DA, Baldwin KD (2019). Approaching unusual pediatric distal humerus fracture patterns. J Am Acad Orthop Surg.

[21] Carrazzone OL, Mansur NSB, Matsunaga FT (2021). Crossed versus lateral K-wire fixation of supracondylar fractures of the humerus in children: a meta-analysis of randomized controlled trials. J Shoulder Elbow Surg.

[22] Kwok SM, Clayworth C, Nara N (2021). Lateral versus cross pinning in paediatric supracondylar humerus fractures: a meta-analysis of randomized control trials. ANZ J Surg.

[23] Queally JM, Paramanathan N, Walsh JC, Moran CJ, Shannon FJ, D'Souza LG (2010). Dorgan's lateral cross-wiring of supracondylar fractures of the humerus in children: a retrospective review. Injury.

[24] Royce RO, Dutkowsky JP, Kasser JR, Rand FR (1991). Neurologic complications after K-wire fixation of supracondylar humerus fractures in children. J Pediatr Orthop.

[25] Topping RE, Blanco JS, Davis TJ (1995). Clinical evaluation of crossed-pin versus lateral-pin fixation in displaced supracondylar humerus fractures. J Pediatr Orthop.

[26] Brauer CA, Lee BM, Bae DS, Waters PM, Kocher MS (2007). A systematic review of medial and lateral entry pinning versus lateral entry pinning for supracondylar fractures of the humerus. J Pediatr Orthop.

[27] Gangadharan S, Rathinam B, Madhuri V (2014). Radial nerve safety in Dorgan's lateral cross-pinning of the supracondylar humeral fracture in children: a case report and cadaveric study. J Pediatr Orthop B.

[28] Omid R, Choi PD, Skaggs DL (2008). Supracondylar humeral fractures in children. J Bone Joint Surg Am.

[29] Parikh SN, Lykissas MG, Roshdy M, Mineo RC, Wall EJ (2015). Pin tract infection of operatively treated supracondylar fractures in children: long-term functional outcomes and anatomical study. J Child Orthop.

[30] Altay MA, Erturk C, Isikan UE (2010). Comparison of traditional and Dorgan's lateral cross-wiring of supracondylar humerus fractures in children. Saudi Med J.

